# A humanized mouse model to study NK cell biology during HIV infection

**DOI:** 10.1172/JCI165620

**Published:** 2022-12-15

**Authors:** Jocelyn T. Kim, Jerome A. Zack

**Affiliations:** 1 Department of Medicine, David Geffen School of Medicine,; 2 Department of Microbiology, Immunology, and Molecular Genetics, and; 3 Department of Medicine, Division of Hematology and Oncology, University of California, Los Angeles, Los Angeles, California, USA.

## Abstract

NK cells are an important subset of innate immune effectors with antiviral activity. However, NK cell development and immune responses in different tissues during acute and chronic HIV infection in vivo have been difficult to study due to the impaired development and function of NK cells in conventional humanized mouse models. In this issue of the *JCI*, Sangur et al. report on a transgenic MISTRG-6-15 mouse model with human IL-6 and IL-15 knocked into the previously constructed MISTRG mice. The predecessor model was deficient in *Rag2* and γ chain (γc) with knock-in expression of human M-CSF, IL-3, GM-CSF, and TPO, and transgenic expression of human SIRPα. The researchers studied tissue–specific NK cell immune responses during HIV infection and clearly show that the endogenous human NK cells in the humanized mouse model suppressed HIV-1 replication in vivo. These findings provide insight into harnessing the innate immune response for clinical antiviral therapies.

## Role of NK cells in HIV infection

NK cells are innate immune effectors capable of intrinsically recognizing and clearing virally infected cells through multiple mechanisms. Epidemiological and genetic studies have shown NK cell interactions with self-HLA molecules are involved in recognition of HIV-infected cells and may slow disease progression, reduce viral setpoint, or mediate immune pressure (1–9). In vitro studies have clearly demonstrated the importance of NK cell interactions with NK cell ligands in recognition of HIV-infected cells (6, 10, 11). In addition to in vitro studies, multiple groups have utilized adoptively transferred NK cells to decrease HIV infection in humanized mice (12–14). However, studying the biology of endogenous human NK cell immune responses during acute and chronic HIV infection in vivo has been limited, due to a shortage of appropriate humanized mouse models.

## Recent humanized mouse models with human NK cells

Humanized NOD.Cg-*Prkdc^scid^ Il2rg^tm1Wjl^*/SzJ (called NSG) mice or B6.129S-*Rag2^tm1Fwa^ Cd47^tm1Fpl^ Il2^rgtm1Wjl^*/J (called TKO) mice are common models used to study acute- and chronic-HIV infection in vivo. However, these humanized mice do not generate robust numbers of human NK cells, making the study of NK cell biology difficult. Two recent mouse models have shown promising development and engraftment of human NK cells into humanized mice. MISTRG mice were previously constructed by knocking in human M-CSF, IL-3/GM-CSF, and TPO into *Rag2^–/–^ γc^–/–^* mice with transgenic expression of human SIRPα. These MISTRG mice demonstrated efficient myeloid cell development as well as improved circulating and tissue–specific NK cell engraftment, particularly in the liver (15, 16). Also, the MISTRG mice have been used to study the dynamics of acute and chronic infection using X4 and R5 tropic HIV isolates (17). However, these humanized MISTRG mice have limited life spans due to severe anemia, possibly from the effects of irradiation and human macrophage-mediated killing of mouse RBCs (15). Clodronate-mediated depletion of human macrophages in HIV-infected MISTRG mice resulted in an increase in viral replication in vivo, despite observing a higher frequency of circulating cells expressing the NK cell–specific marker NKp46 (17). Thus, whether endogenous human NK cells could control HIV replication in these mice was unclear. Next, the SRG-15 mice were developed by knock-in replacement of human IL15 and human SIRPα into a Rag2^–/–^ Il2rg^–/–^ mice to generate physiological tissue expression of human IL-15 (18). Humanized SRG-15 mice demonstrated circulating and tissue-specific NK cells capable of mediating antibody-dependent cellular toxicity (ADCC) in vivo using anti-CD20 monoclonal antibody against a xenograft B cell tumor challenge (18). Recently in SRG-15 mice, ADCC function played an important role in decreasing HIV replication, the viral reservoir, and viral rebound in animals treated with a combination of a CD4-mimemtic compound and CD4-induced antibodies, which stabilized the HIV envelope in a conformation conducive to NK cell targeting by ADCC (19).

## NK cells generated in humanized MISTRG-6-15 mice

**Figure 1:**
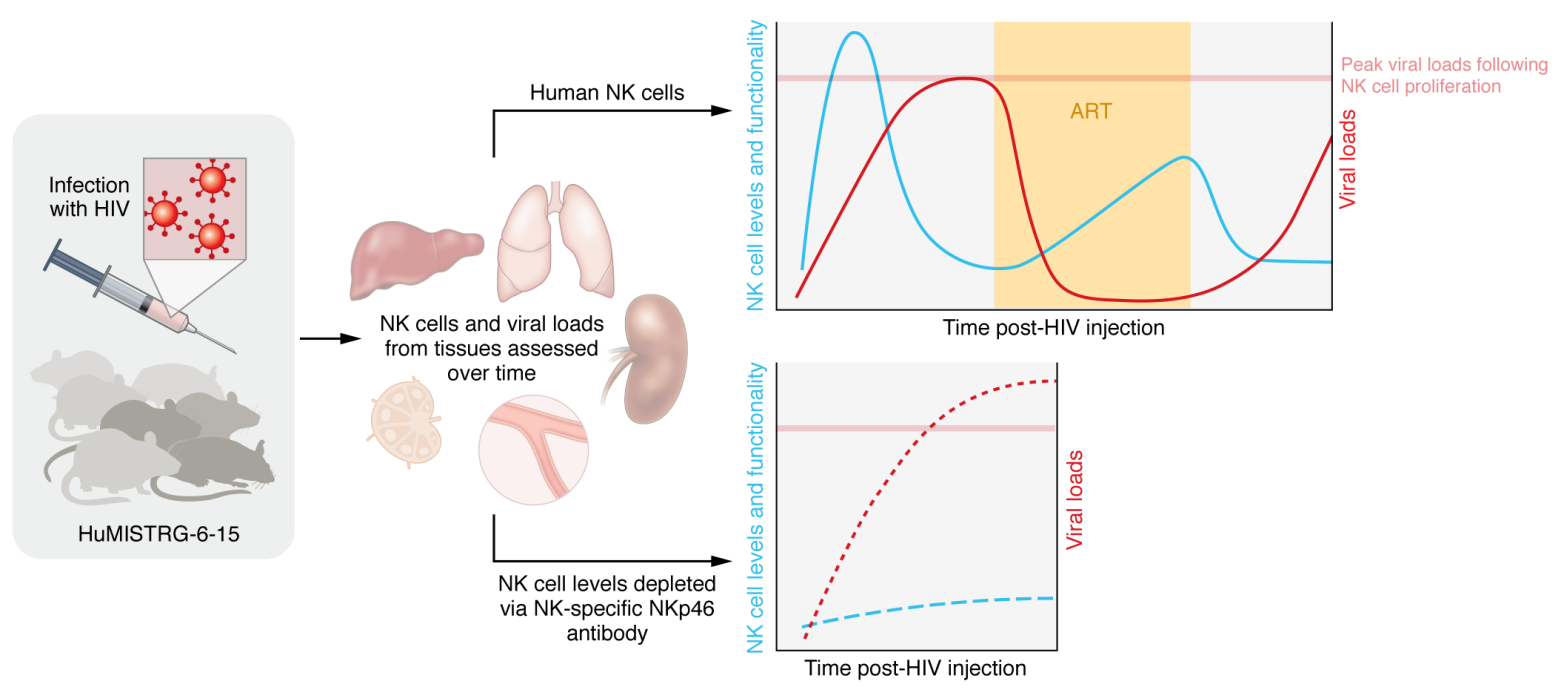
Endogenous human NK cells suppress HIV-1 replication in HIV-infected humanized MISTRG-6-15 mice. Sungur and colleagues followed NK cells longitudinally from specific tissues, including blood, liver, spleen, lungs, and lymph nodes. NK cell functionality varied during the course of acute and chronic HIV infection, ART treatment, and viral rebound after ART discontinuation. NK cells demonstrated increased activation, proliferation, and functionality during acute infection, but then showed reduced functionality and immune exhaustion during chronic infection. ART only partially restored NK cell levels and functionality compared with animals that rebounded after ART interruption. Importantly, viral levels increased during acute infection if mice were depleted of NK cells via an NK-specific NKp46 antibody, indicating that NK cells directly suppress HIV-1 replication in vivo (20). HuMISTRG-6-15, humanized MISTRG-6-15.

In this issue of the *JCI*, Sangur and colleagues created MISTRG-6-15 mice by knocking in MISTRG mice with human IL-6 and IL-15 (20). After transplantation of hematopoietic stem and progenitor cells (HSPCs) obtained from human cord blood, these mice showed improved human NK cell repopulation compared with the commonly used humanized NSG mouse model. The NK cells in MISTRG-6-15 mice were quick to expand, and, upon HIV infection, nonlymphoid organs exhibited degranulation, cytotoxicity, and cytokine production. Furthermore, the NK cells in lymphoid organs had reduced CD16 expression and functionality, which could reflect similar tissue-specific differences found in human circulating and tonsillar NK cells. One important strength of this study was the ability of the authors to follow HIV infection in MISTRG-6-15 mice for over five months while longitudinally sampling NK cells and performing ex vivo functional tests (Figure 1). The NK cells collected during the first weeks of acute infection demonstrated increased activation, proliferation, and functionality ex vivo. In comparison, NK cells sampled during the several months after initial infection demonstrated immune exhaustion, shown by an increase in immune checkpoint–receptor surface expression and a decrease in ex vivo functionality. Viral replication was suppressed in vivo with antiretroviral treatment (ART), which then partially restored NK cell levels and functionality compared with animals exhibiting rebound viremia after ART interruption (20).

Most importantly, the authors convincingly showed that NK cell depletion mediated by a NK-specific NKp46 antibody resulted in increased plasma and tissue cell–associated HIV-1 RNA levels (20). This result indicates that circulating and tissue-specific NK cells directly suppressed HIV-1 replication in vivo. The finding is also consistent with our recent results showing that the addition of exogenous human NK cells limits viral rebound following cessation of ART in a different humanized mouse model (14). The authors also utilized a broadly neutralizing antibody (bNab) — PGT121, with a mutation to disrupt Fc binding — to show that NK activation and functionality was enhanced in an Fc-dependent manner (20), which is consistent with a recent study showing that NK cell ADCC function in SRG-15 mice can be harnessed to control and reduce HIV infection (19).

Sangur and colleagues predicted that knock in of human IL-6 would create a more physiologically relevant mouse model compared with NSG mice humanized with cord blood CD34^+^ HSPCs. They suggested that human IL-6 expression stimulates human HSPC and myeloid differentiation, while partially blocking murine hematopoiesis. In addition, they suspect that knock in of human IL-15 improved NK engraftment in their mouse model (20). However, it remains unclear whether humanized MISTRG-6-15 mice are superior to more recent humanized MISTRG or SRG-15 mice, as direct comparisons were not performed.

The authors tackle an exciting area of research in studying the innate immune response during HIV infection (20). The MISTRG-6-15 mice will be important to elucidate which NK receptor and ligand interactions are required for recognition and clearance of HIV-infected cells in vivo in future studies. This model will also facilitate the development of strategies to harness the innate immune response against HIV infection.

## Version 1 12/15/2022

Electronic publication
